# Non-Targeted Metabolomics Investigation of a Sub-Chronic Variable Stress Model Unveils Sex-Dependent Metabolic Differences Induced by Stress

**DOI:** 10.3390/ijms25042443

**Published:** 2024-02-19

**Authors:** Seulgi Kang, Woonhee Kim, Jimin Nam, Ke Li, Yua Kang, Boyeon Bae, Kwang-Hoon Chun, ChiHye Chung, Jeongmi Lee

**Affiliations:** 1 School of Pharmacy, Sungkyunkwan University, Suwon 16419, Republic of Korea; seulgi0415@skku.edu (S.K.); ke.lee@outlook.com (K.L.); dneyngos@skku.edu (Y.K.); kuy04209@skku.edu (B.B.); 2 Department of Biological Sciences, Konkuk University, Seoul 05029, Republic of Korea; iamwoonhee@gmail.com (W.K.); skawlals05@gmail.com (J.N.); cchung@konkuk.ac.kr (C.H.C.); 3 Gachon Institute of Pharmaceutical Sciences, College of Pharmacy, Gachon University, Incheon 21936, Republic of Korea; khchun@gachon.ac.kr

**Keywords:** sex-dependent depression, metabolomics, rodent stress model, energy metabolism, hypothalamic-pituitary-adrenal axis

## Abstract

Depression is twice as prevalent in women as in men, however, most preclinical studies of depression have used male rodent models. This study aimed to examine how stress affects metabolic profiles depending on sex using a rodent depression model: sub-chronic variable stress (SCVS). The SCVS model of male and female mice was established in discovery and validation sets. The stress-induced behavioral phenotypic changes were similar in both sexes, however, the metabolic profiles of female plasma and brain became substantially different after stress, whereas those of males did not. Four stress-differential plasma metabolites—β-hydroxybutyric acid (BHB), L-serine, glycerol, and myo-inositol—could yield biomarker panels with excellent performance to discern the stressed individuals only for females. Disturbances in BHB, glucose, 1,5-anhydrosorbitol, lactic acid, and several fatty acids in the plasma of stressed females implied a systemic metabolic shift to β-oxidation in females. The plasma levels of BHB and corticosterone only in stressed females were observed not only in SCVS but also in an acute stress model. These results collectively suggest a sex difference in the metabolic responses by stress, possibly involving the energy metabolism shift to β-oxidation and the HPA axis dysregulation in females.

## 1. Introduction

Depression is envisaged as a result of complex interactions between environmental and biological factors [1], and is now recognized as the number one cause of disability worldwide [2]. Depression is a huge health concern and a compounding factor that predisposes patients to other diseases such as cardiovascular diseases [3]. Notably, women are clinically twice as susceptible to depression compared to men [1]. Numerous preclinical models have been employed to understand the mechanisms underlying the pathophysiology of depression [4,5,6,7], however, males have been exclusively studied in most cases [8]. Several sex difference factors have been postulated to be associated with sex-dependent depression, including hormone fluctuations and the hypothalamic-pituitary-adrenal (HPA) axis [1]. Nonetheless, the detailed underlying mechanisms of varying susceptibilities to depression between men and women remain largely unknown [9], necessitating careful investigation of appropriate models for accurate comprehension of the pathophysiology and treatment of depression depending on sex [8].

There are a limited number of approaches to modelling aspects of sex differences in depression. The female animal models of depression are based on several categories of inducing manipulation, including stress (physical and psychological), pharmacology, and pathology [9]. Examples of the physical stress-based rodent models include chronic learned helplessness (cLH), chronic unpredictable mild stress (CUMS), and chronic variable stress (CVS) models [8]. CVS for 21 to 28 days generally employs more intense stress with a less variable schedule than CUMS, and the stress period can be shortened to 6 days or less, which is then called sub-chronic variable stress (SCVS) [8]. The SCVS model was used to study the mechanisms for depression in females on the transcriptome [10] and neural circuit [11] levels. Concerning depression-like behaviors, SCVS was reported to induce decreased sucrose preference [11] and decreased grooming in the splash test only in female mice [10]. However, sex convergence in the behavioral phenotypes was also reported despite sex-specific molecular changes in the SCVS model [12].

Mass spectrometry (MS)-based non-targeted metabolomics studies serve as routine and unbiased platforms that provide insights into biomarkers to pathways and mechanisms [13]. Our previous studies on the CUMS models revealed that depression-induced male mice underwent significant biochemical changes in biological fluids [14,15], although females were not studied. Sex-associated traits in various diseases have been studied by the metabolomics-based investigation of preclinical and clinical samples. For example, sex-specific differences in energy metabolism were observed in the brain of type 1 diabetic mice [16], and mouse models of Alzheimer’s disease showed sex-dependent differences in amino acid, carbohydrate, and fatty acyl metabolisms in the brain [17]. Regarding depression, plasma metabolites [18] or a panel of urinary metabolites [19] were suggested as sex-dependent diagnostic biomarkers in humans. Sex-dependent changes in plasma phosphatidylcholines and sphingomyelins were reported based on the metabolomics and lipidomics investigation of a sub-chronic restraint stress rat model [20].

This study aimed to examine whether stress could induce sex-dependent metabolic changes in plasma and the brain in an SCVS model. To this end, both female and male mice were used, and their plasma and the whole brain were analyzed using gas chromatography (GC) MS and liquid chromatography (LC) MS platforms for non-targeted metabolomics investigation. As a result, numerous sex- and stress-differential metabolites and metabolic pathways were deduced from two independent experimental sets, and a valid panel of diagnostic biomarkers for stressed females was constructed. Subsequently, several key biomolecules were quantified in the SCVS model to test the metabolomics-driven hypothesis. This non-biased study provides insights into how females and males express metabolic changes differently under the same stress.

## 2. Results

### 2.1. SCVS Induced Changes in Behavioral Phenotype in Female and Male Mice

**Figure 1:**
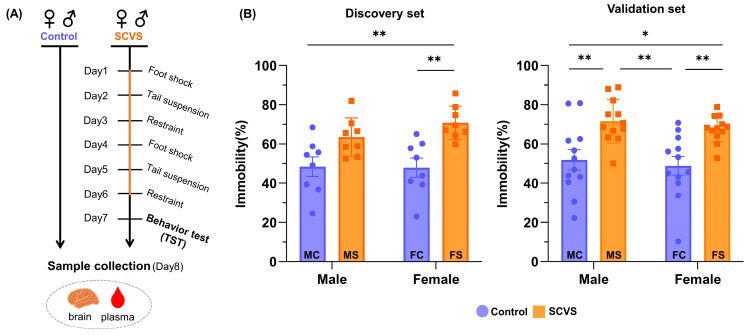
Scheme for the establishment of SCVS model in the discovery and validation sets (**A**) and tail suspension test results (**B**). * and ** indicate significant differences with *p* < 0.05 and *p* < 0.01, respectively. Results in (**B**) are displayed as mean ± S.E.M. Number of mice per group: n = 8 in the discovery set and n = 12 in the validation set.

The SCVS model was established for both sexes using the stress regime in Figure 1A. Each set of models included four groups of mice: females without stressors (FC), females with stressors (FS), males without stressors (MC) and males with stressors (MS). In a preliminary study, behavioral phenotypes of mice were characterized using an open field test (OFT), novelty-suppressed feeding test (NFS), and tail suspension test (TST). There appeared to be no sex differences in the locomotor activity (no significant changes in both sexes in the OFT) and the latency to feed (significant increases in both sexes in NSF) upon stress, however, the immobility time increase in TST appeared to be female-specific. Subsequently, two independent sets in which only TST was used as the behavior test were established for the non-targeted metabolomics analysis of the plasma and brain. The first set was intended for the biomarker discovery and the second set was for validation. Figure 1B shows that the average basal immobility times of the male and female control mice (MC and FC) were similar, with large individual variations within groups in both discovery and validation sets. In comparison, the stressor treatment increased the immobility times with narrower variations in both sexes (MS and FS). Specifically, the FS mice showed significant increases in both sets (*p* < 0.01). The increases in MS were statistically significant only in the validation set (*p* < 0.01).

### 2.2. Metabolic Profiles of the Plasma and Brain Were Different Depending on Sex and Stress

The discovery set of the SCVS model was applied to the non-targeted metabolomics analysis to examine the metabolic changes associated with sex difference and stressor treatment. All the datasets from the GC-MS and LC-MS analyses of the discovery set (brain and plasma samples) were pre-processed and applied to PCA, resulting in the score plots in Figure S1. The GC- and MS-based profiles of the plasma (Figure S1A) and brain (Figure S1B) samples showed that the FS mice tended to cluster compared with the other three groups of mice. In contrast, the LC- and MS-based profiles of the brain (Figure S1C,D) showed no noticeable clustering tendencies within the groups. After confirming that there were no apparent outliers in the samples, all the datasets were used for the supervised analysis by OPLS-DA. Numerous classification models were built, however, only the models with Q^2^ values >0.3 were considered reasonable and processed for the subsequent cross-validation by permutation test to ensure non-overfitting (n = 100; Figure S2).

Seven valid differential models were established in total, and the model quality parameters are summarized in Table 1. The GC- and MS-based profiles yielded six differential models: five from the plasma and one from the brain. The results indicate that the plasma contained many more discriminant variables than the whole brain upon GC-MS analysis. The plasma-derived models included four binary classification models (FC vs. MC, FS vs. FC, FS vs. MS, and FS vs. the others) and one multi-classification model (FS vs. FC vs. males). The brain-derived model was from FS vs. FC. In the case of LC-MS analysis, plasma profiles were unobtainable because of the limited plasma volume, and only one discriminant model (FS vs. FC) was established from the brain samples. The male mice of the discovery set yielded no valid stress-differential models in either the plasma or brain.

### 2.3. The SCVS Model Revealed Sex- and Stress-Differential Metabolites and Metabolic Pathways

Differential metabolites for sex and stress were extracted from the discovery set. The numbers of metabolites selected in the differential models are summarized in Table 1, for which only the metabolites with AUC ≥ 0.7 were considered. Although metabolic sex differences have been reported in the mouse brain [21], no valid sex-differential model was available in our study of the brain. In contrast, plasma clearly displayed sex differences as represented in the 25 sex-differential metabolites. Among the female-specific plasma metabolites identified are L-serine, L-lysine, and citric acid, which were all upregulated by approximately 2–10 fold in female mice compared with male mice (Table S1).

**Figure 2:**
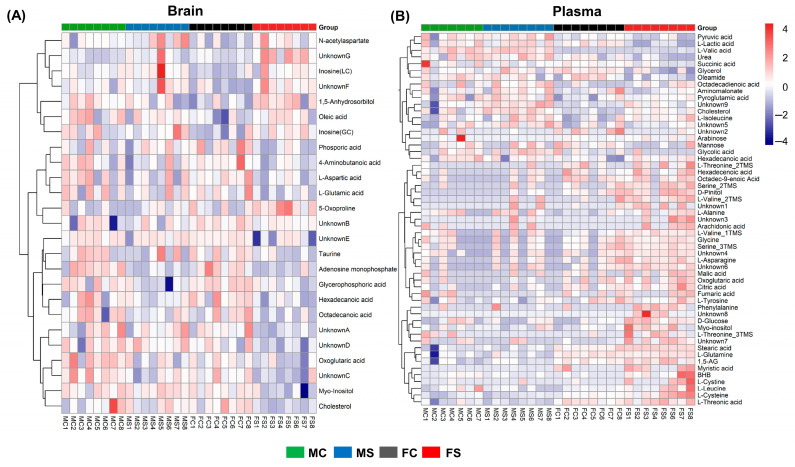
Hierarchical clustering analysis and heat map of the selected differential metabolites in brain (**A**) and plasma (**B**). Group identification: FC, female without SCVS; FS, female with SCVS; MC, male without SCVS; MS, male with SCVS.

**Figure 3:**
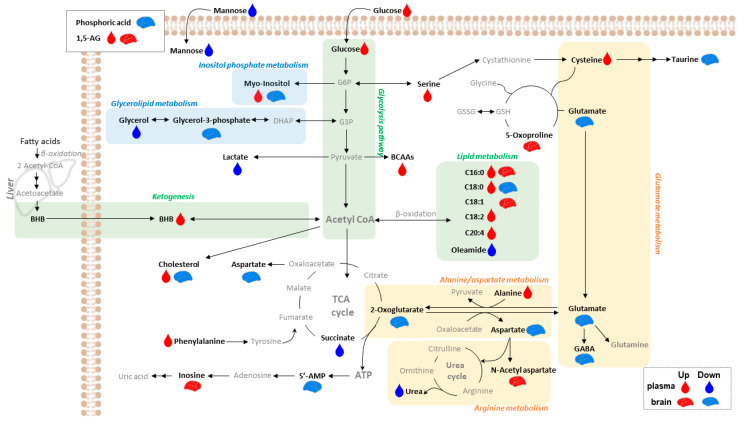
Overview of the integrated metabolic disturbances in the SCVS-treated female mice. Abbreviations: BCAA, branched chain amino acid; G6P, glucose-6-phosphate; G3P, glyceraldehyde-3-phosphate; 1,5-AG, 1,5-anhydroglucitol; BHB, 3-hydroxybutyric acid; C16:0, palmitic acid; C18:0, stearic acid; C18:1, oleic acid; C18:2, linoleic acid; C20:4, arachidonic acid.

After the SCVS treatment, the female presented 23 stress-specific brain metabolites (FS vs. FC, Table S2). In plasma, the female showed 41 stress-specific metabolites, which include 22 stress-differential metabolites among the female mice (FS vs. FC) and 39 sex-differential metabolites among the stressed mice (FS vs. MS) (Table 2). Many of these sex-differential metabolites overlap with the stress-differential metabolites with significant fold changes in the same direction, including L-lactic acid, L-alanine, β-hydroxybutyric acid (BHB), urea, L-serine, phenylalanine, glycerol, D-glucose, and oleamide. All the differential brain and plasma metabolites were further applied to the hierarchical clustering analysis (HCA), of which the results are illustrated in Figure 2. Many of the differential brain metabolites showed downregulation in the FS mice (Figure 2A). The plasma of the FS mice presented unique signatures, which were characterized primarily by upregulation compared with those of the other groups of mice (Figure 2B). All the identified differential metabolites were used for the pathway analysis to examine the perturbed metabolic pathways in the SCVS-treated female mice. The most significantly affected pathways with impact values >0.1 and –log10(*p*) >2.0 include alanine, aspartate, and glutamate metabolism and arginine biosynthesis in the brain. A schematic of the affected pathways based on biochemical relationships is presented in Figure 3.

### 2.4. Biomarker Panels Distinguished the Metabolic Status Only between Stressed and Unstressed Female Mice

A scheme to construct a plasma biomarker panel is outlined in Figure S3A. First, the 41 plasma metabolites in the discovery set (Table 2) were used as the starting pool. Then, the differential metabolites against both FC and MS with AUC ≥ 0.8 were screened, and large fold changes (>2 or <0.5) were deemed preferable. Meanwhile, the validation set was also subjected to extraction of differential metabolites, of which relative changes and AUC values were also assessed (Tables S3 and S4 for brain and plasma, respectively). Of note, the male mice in the validation set presented discernable metabolic profiles neither in the brain nor in plasma upon SCVS, although their TST immobility time increased significantly as a result of stress. The biomarker candidates from the discovery set were further compared with those from the validation set to reveal metabolites that consistently showed remarkable changes in the same trends in both sets. As a result, the following four metabolites were selected as the biomarkers for differentiating the stressed subjects among the female mice: BHB, L-serine, glycerol, and myo-inositol.

Three prediction models of the biomarker panels were constructed using three machine learning algorithms: support vector machine (SVM), random forest (RF), and logistic regression (LR). For example, the LR model is as follows: prediction probability = e^y^/(1 + e^y^), y = 0.342 + 1.3078(BHB) − 0.1607(myo-inositol) + 0.8205(glycerol) + 0.6959(L-serine). All three biomarker panels generally showed good diagnostic performance for the female mice in the test set (accuracy, 0.82−1.00; sensitivity, 0.60−1.00; specificity, 1.00) and the validation set (accuracy, 0.91–1.00; sensitivity, 0.83–1.00; specificity, 1.00) (Table S5). Figure S3B–D displays the ROC curves of the three models. Their AUC values for FS vs. FC in the training set ranged between 0.943−0.967, and the test and validation sets yielded 1.000 in all the models. In contrast, the models showed poor discrimination power for the male mice, as seen in the low AUC values (0.446−0.646) in both the discovery and validation sets.

### 2.5. Only Females Displayed Disturbances in Glucose Metabolism and the Hypothalamic-Pituitary-Adrenal Axis after Stress

The non-targeted metabolomics analysis above suggested disturbances in energy metabolism induced by SCVS in females. Here, the plasma levels of relevant molecules, BHB, glucose, and insulin were quantified. The plasma levels of corticosterone were also measured. Only the discovery set, for which mice were ad libitum-fed, could be analyzed for this measurement because the plasma volume in the validation set was insufficient.

**Figure 4:**
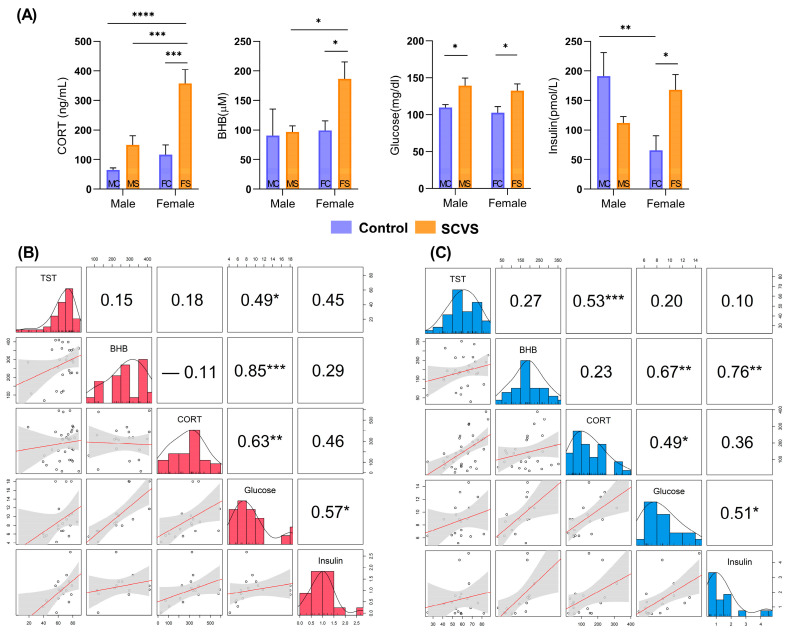
(**A**) Plasma concentrations of key biomolecules in the discovery set of SCVS model. Data are shown as mean ± S.E.M (n = 8 per group). (**B**) Correlation analysis among female mice. (**C**) Correlation analysis among male mice. Numbers and red lines in box (B and C) indicate the correlation coefficients and regression lines, respectively. *, **, ***, and **** indicate *p* < 0.05, *p* < 0.01, *p* < 0.001, and *p* < 0.0001, respectively. Abbreviations: CORT, corticosterone; BHB, 3-hydroxybutyric acid.

The relative differences in glucose and BHB concentrations (Figure 4A) generally agreed with the metabolomics results. The glucose concentrations in FS and MS significantly increased, while the insulin levels increased significantly in FS. The BHB concentration was significantly elevated in the FS mice compared with that of the other groups (e.g., FS vs. FC, *p* < 0.05; FS vs. MS, *p* < 0.05). Before stress, the female mice had higher baseline levels of corticosterone than the male mice. The corticosterone concentrations were then significantly elevated by SCVS only in the females (FS vs. FC, *p* < 0.001). According to the Spearman correlation analysis (Figure 4B,C), the corticosterone level was correlated with the immobility time in males (r = 0.53, *p* < 0.005) but not with that of females (r = 0.18, *p* > 0.05). The corticosterone level showed a positive correlation with the glucose levels in both males (r = 0.49, *p* < 0.05) and females (r = 0.63, *p* < 0.01), however, no significant correlation was observed between the corticosterone and BHB levels in males (r = 0.23, *p* > 0.05) or females (r = −0.11, *p* > 0.05).

## 3. Discussion

Our study reports the non-targeted metabolomics investigation of the SCVS model, one of few pre-clinical models to study sex differences in depression. Various behavioral tests have been used to assess stress susceptibility in the SCVS model, including sucrose preference, NSF, forced swimming, and social interaction tests that measure anhedonia, anxiety-like, despair, and social behaviors [1,22]. The limitation is that their validity has yet to be confirmed for female rodents [9]. Initially, OFT and NSF tests were included in the preliminary experiment. Given with no apparent sex difference in the anxiety-like phenotypes by these tests, they were omitted in the subsequent sets in order to minimize the time gap between the last stressor of SCVS and the sampling. Regarding despair behavior, immobility time in TST was measured in this study. TST was originally introduced to evaluate the therapeutic effects of potential antidepressants in mice [23], and was also used as a measure of depressive behavior in a depression model [24]. FS and MS showed statistically different immobility increases in the discovery and validation sets. Thus, it was inappropriate to determine whether the SCVS induced sex difference or sex convergence in the depression-like phenotype. In this regard, no sex difference was observed in the FST results in a five-day sub-chronic stress model in which forced swimming was included twice as the stressor [12]. Although the TST was performed one day after the last stressor (restraint), habituation to tail suspension might have been reflected in our results as suggested previously [12]. The ideal way to conduct TST would be to compare the immobility times of the same subject before and after SCVS (in that case, metabolome data of the mice before SCVS is unobtainable because the brain sampling requires sacrifice of the subject). Hence, the averaged immobility times of control and stressed groups had to be compared in our study. In the discovery and validation sets, FC and MC displayed relatively large individual variations of immobility times. Based on the varying basal abilities to cope with tail suspension in both sexes, mice were divided into high- and low-immobility groups before establishing a depression model, and TST was again applied to assess depressive-like behavior [25]. Therefore, the large immobility time variations in our study may reflect innately different sensitivities to tail suspension. Future studies of SCVS may benefit from employing a higher number of mice for stratification according to the basal immobility time of TST. More importantly, multiple tests are desirable for the comprehensive assessment of depressive behavioral phenotypes [26].

Regardless of the TST results, we observed significant metabolic changes in the plasma and brain of female mice but not in those of male mice. With regard to sex differentiation, L-serine, L-lysine, and citric acid in the FC mice were upregulated compared with the MC mice. All of these metabolites have been previously reported as the sex-differential markers in human serum/plasma or urine, with their relative levels being higher in females than in males [27]. Our results support the validity of the three metabolites as the sex-differential plasma biomarkers in humans and mice. Intriguingly, L-serine was also identified as an FS-specific plasma marker against FC as well as against MS. In line with this, the serum serine level was elevated in patients with depression [28], and the association of glycine, serine, and threonine metabolism with a female-specific mood disorder was suggested in a clinical urine metabolomics study [29].

The FS brain levels of neurotransmitters, 4-aminobutanoic acid (GABA) and L-glutamic acid, were consistently low compared with FC in both the discovery and validation sets. L-glutamic acid was identified as the biomarker in the brain of the male CUMS model [30] and in the prefrontal cortex of the male lipopolysaccharide-induced depression model [5], while GABA was identified as the biomarker in the hippocampus of the male CUMS model [31]. Therefore, it is presumable that alterations in the glutamatergic and GABAergic systems in the brain are the common players associated with depression independent of sex [32]. Several lipid metabolism-related metabolites were altered in the FS mice. For example, stearic and oleic acids, glycerol, and myo-inositol were increased in the plasma, while cholesterol was increased in plasma but decreased in the brain compared with FC. Disturbances in lipid metabolism has been associated with depression in both human patients [33] and male animal models [5,6].

SCVS induced the most noticeable difference in plasma BHB levels in a sex-dependent manner based on the metabolic profiles. The relative level of BHB in FS to FC and MS was above an order of magnitude. Conversely, there was no significant difference between MS and MC. BHB is a ketone body as a product of fatty acid oxidation in the liver. Besides being an intermediate metabolite, BHB has diverse regulatory roles and therapeutic and diagnostic potentials for various diseases [34]. The plasma BHB showed a positive correlation with depression in humans [35,36]. Male mice had increased blood BHB levels after 1-h restraint stress. Based on the positive correlation between depressive behaviors and BHB levels in a social defeat model, the authors suggested BHB as the indicator of stress vulnerability and neuroinflammation [37].

The discovery of reliable biomarkers for the diagnosis, treatment, and pathophysiology of depression in humans has long been pursued [38]. The numerous metabolites suggested from biological fluids include kynurenic acid [39], laurylcarnitine [40], biliverdin [18], tryptophane, lysophosphatidylcholines [41], citrate, and succinate [19], many of which were discovered without the sex consideration. Biochemical diagnoses of depression in the preclinical model is also worth investigating in favor of avoiding the time-consuming behavior tests and future translation to clinical biomarker discovery. As a biomarker panel rather than a single biomarker can better diagnose and predict diseases [42], we constructed three plasma biomarker panels comprising four metabolites in each panel using different machine learning algorithms. Our study is the first presentation of the biomarker panels that could discern the metabolic status between stressed and unstressed in only females with excellent diagnostic performance. The biomarker panels may be worth further investigation in translational research.

The plasma metabolic signatures of the FS mice in comparison with FC and MS could be sketched by the upregulation of several metabolites including BHB, glucose, 1,5-anhydrosorbitol or 1,5-anhydro-D-glucitol (1,5-AG), glycerol, and several free fatty acids. This observation led us to infer that the stressed females experienced glucose metabolism disturbance with increased lipolysis and lipid metabolism. Energy metabolism imbalance has been commonly reported in various neurological disorders [43]. Recent targeted metabolomics-based studies of CUMS male rats proposed that the susceptibility to chronic stress in male rats is associated with how the brain energy is supported [43,44]. Specifically, the metabolic signatures of the ventral hippocampus in stress-vulnerable animals indicated an energy metabolism shift toward fatty acid utilization [43]. Ketone bodies such as BHB are the only energy providers for the brain besides glucose, and BHB is a marker of lipid metabolism favored over glucose metabolism [45]. Therefore, the stress-induced metabolic disturbance in female mice is proposedly associated with a systemic energy metabolism shift to β-oxidation. Indeed, the plasma concentrations of BHB increased significantly only in the SCVS-treated females.

Glucocorticoids, which exert metabolically opposing effects to insulin, are the allostatic mediators related to metabolic dysregulation [46] and were proposed to link insulin sensitivity and depression in male mice [47]. The sex dimorphism in the HPA axis is known to be responsible, in part, for sex-dependent depression in humans and rodents [48]. The HPA axis dysregulation caused by chronic stress is usually more pronounced, with greater corticosterone increases in females than males [49]. Our corticosterone results support the existence of a sex difference in the HPA axis regulation after sub-chronic stress; female rodents were more prone to develop HPA axis dysregulation than males. The corticosterone levels of female rodents in chronic stress models generally had a weak correlation with depressive behaviors [50]. In this vein, the TST immobility time positively correlated with the corticosterone level in only males, however, immobility could not be interpreted as a direct measure of depressive behavior as discussed above. Glucocorticoids can activate peripheral tissues to mobilize energy stores and induce lipolysis [48]. The positive correlation of the corticosterone level with the glucose level, but not with the BHB level, in both sexes suggests that the stress-induced HPA axis activation is likely to contribute to the elevation of glucose-mediated energy availability in both sexes. It also suggests that the metabolic shift to β-oxidation upon stress in females is not likely in direct association with the HPA axis dysregulation.

One thing to note is that a portion of the FS-specific metabolites discussed above were identified as stress-differential metabolites in the male depression models. Most previous models were based on chronic stress (usually CUMS) from which brain sub-regions such as the hippocampus were analyzed [15,51]. In the recent study of a six-week CUMS male model, the stress-vulnerable rats presented the metabolic shift to β-oxidation in the brain compared with the stress-resilient rats [43]. Therefore, it is unknown whether the sex difference in the metabolic changes in our SCVS model will be repeated in other stress models. In other words, it remains to be revealed whether the females developed such metabolic changes earlier than the males did in a relatively short stress regime in this study. The biological samples were taken one day after the TST to allow the mice to recover from the tail suspension stress. In comparison, samples were collected right after the behavior tests in studies where the stressed males presented distinct metabolic profiles from the unstressed males, although chronic stress regimes were still used [5,15,31]. Thus, relatively subtle changes in the stressed males might have been offset or recovered before the sampling. In this context, the male mice appeared metabolically more resilient to SCVS than the female mice. For the brain, it is also possible that the whole brain analysis blunted metabolic changes in various sub-regions caused by the SCVS. Future analysis of region-specific brain samples of SCVS and immediate sampling after less stressful behavior tests could help to clarify the issues above.

In conclusion, our non-targeted metabolomics investigation of the SCVS model reveals the sex difference in the metabolic changes upon stress. Our study provided well-performing diagnostic biomarker panels which could metabolically discern the stressed from the unstressed among female mice. It was suggested that the stressed female mice undergo the energy metabolism shift towards β-oxidation. It was also found that the stress induced the HPA axis hyperactivation in females, while the HPA axis dysregulation was not directly associated with the metabolic shift. Our non-biased or bottom-up study underpins the underlying mechanisms suggested for different metabolic responses to stress within the same sex and between different sexes in previous studies. Women are more vulnerable to stress-related psychopathologies than men. Preclinical and clinical evidence suggests that stress exposure potentially contributes as a causal factor in the development of depression [52]. Therefore, the current study provides a clue to elucidate the intermediate links between stress exposure and response and the occurrence of depression in women.

## 4. Materials and Methods

### 4.1. SCVS Model

Male and female C57BL/6J mice aged 10 weeks were purchased from Orient Bio (Seongnam, Korea). All mice were maintained and treated after approval from the Institutional Animal Care and Use Committee of Konkuk University.

For the SCVS model, mice were randomly divided into two control groups (male control, MC; female control, FC) and two stressed groups (male SCVS-treated, MS; female SCVS-treated, FS). SCVS comprising two repeated cycles of three alternating stressors (6 days in total) was implemented as previously described with slight modifications [10]. A scheme of the SCVS and subsequent animal experiments is displayed in Figure 1A, and the detailed procedures are described in Experimental S1 in ESI. The SCVS model, in which mice had free access to food until sampling, was independently established twice for the non-targeted metabolomics investigation: one for the marker discovery (discovery set; n = 8 per group) and the other for the marker and biomarker panel validation (validation set; n = 12 per group).

### 4.2. Behavior Tests and Sample Collection

A tail suspension test (TST) was performed approximately 24 h after the last stressor (on day 7 for the SCVS model), which was between 10am and 12pm. Blood and brain samples were collected 24 h after the behavior test. For TST, after habituation for 1 h, each mouse was treated as previously described under white noise conditions [53]. Their behaviors were recorded for 6 min, and the total sum of immobility periods was measured by open-source software Bonsai Version 2.6.3 (Open Ephys, Atlanta, GA, USA). The results were analyzed by one-way ANOVA, followed by Fisher’s LSD post hoc test using Prism 7.0.

For sampling, the mice were subjected to isoflurane inhalation. Blood was drawn through a cardiac puncture and collected in a lithium heparin tube. After blood collection, the mice were decapitated, and their whole brains were transferred into conical tubes. Plasma was isolated by centrifuging the blood at 1500× *g* at 4 °C for 10 min. The collected plasma and brain samples were immediately aliquoted and frozen with liquid nitrogen and then stored at −80 °C until further analysis.

### 4.3. Sample Preparation for the Non-Targeted Metabolic Profiling

Aliquots of plasma and brain were thawed on ice before sample preparation. Plasma (150 μL) was mixed with three volumes of acetonitrile and 30 μL of an internal standard solution containing alanine-d4, 4-choloro-DL-phenylalanine, and stearic acid-d35 at 100 μg mL^−1^ for each was added. After vortexing for 30 s, the mixture was centrifuged at 14,680× *g* at 4 °C for 10 min, and the cleared supernatant was collected and dried. A portion of the weighed brain was mixed with methanol at 1:10 (*w*/*v*) and extracted using a homogenizer (IKA^®^, Boutersem, Belgium). After centrifugation at 14,680× *g* at 4 °C for 10 min, the supernatant was collected and dried, and the internal standards (ribitol, alanine-d4, stearic acid-d35 for GC-MS, and 4-choloro-DL-phenylalanine and chlorpropamide for LC-MS) were spiked in the supernatant. Quality control (QC) samples were prepared by pooling an equal volume of every sample. 

For the GC-MS analysis of plasma and brain samples, the derivatization conditions and the scheme for batch preparation and analysis were optimized to acquire as many metabolite peaks as possible while ensuring decent stability of the derivatives (Figure S4). Details on this procedure are provided in Experimental S2 and Figure S5. The brain extract was also analyzed using an ultra-high performance liquid chromatography coupled with a quadrupole-time-of-flight mass spectrometry (UHPLC-Q-TOF-MS) system [54]. Due to limited volume, the plasma samples could not be analyzed by UHPLC-Q-TOF-MS. The operational details are provided in S3.

### 4.4. Data Pre-Processing, Chemometric Analysis, and Data Interpretation

The acquired data were pre-processed using Markerlynx XS ver. 4.1 software for chromatographic peak picking, MS spectral deconvolution, baseline correction, alignment of retention time (RT), mass (*m*/*z*), and normalization [55,56]. Each dataset was independently normalized using internal standards. The pre-processed datasets were Pareto-scaled to give equal weight to the variable [57] before multivariate statistical analyses were conducted using SIMCA-P ver. 11.5 (Umetrics AB, Umea, Sweden), by which principal component analysis (PCA) and orthogonal projection to latent structures discriminant analysis (OPLS-DA) were performed. The quality and reliability of the models were assessed based on the cumulative modeled variation (R^2^X and R^2^Y), cross-validated predictability (Q2) values, and the 100-iteration permutation test [58]. The variable importance in the projection (VIP) values >1 were filtered out as discriminant variables, for which independent Student’s *t*-test and false discovery rate (FDR) by the Benjamini and Hochberg method were further applied (*p* < 0.05 and q < 0.2) using Prism (GraphPad Software 7.0, Inc., Boston, MA, USA).

The dataset from SIMCA-P was put into MetaboAnalyst 5.0 (https://www.metaboanalyst.ca; accessed on 21 June 2021) for pathway analysis and interpretation. Pathway analysis of the identified metabolites was performed according to the KEGG database. A heat map was constructed based on the Z-score as previously described [54], and the hierarchical clustering analysis (HCA) was performed using the Ward method with Euclidean distance. R-package ‘pROC’ was used for receiver operating characteristic (ROC) analyses, and the predictive capability of discriminant metabolites was evaluated based on the area under the curve (AUC) [59]. Machine learning algorithms were run in R to establish a biomarker panel of stressed subjects after hyperparameter tuning. The ‘random forest’ and ‘kernlab’ for RF and SVM, respectively, were applied in R-packages, and ‘glm’ for LR in the R-function. The model performance was evaluated based on AUC, specificity, sensitivity, classification accuracy, and precision as previously defined [60]. Spearman’s correlation analysis was conducted using ‘corrplot’ in R-packages.

### 4.5. Biochemical Assays to Quantify the Concentrations of Key Biomolecules

The plasma levels of glucose and 3-hydroxybutyric acid or β-hydroxybutyric acid (BHB) were measured using commercial kits MAK263-1KT and MAK041, respectively, both of which are from Sigma-Aldrich. The plasma concentrations of corticosterone (Cat#EIACORT, Thermo Fisher, Waltham, MA, USA) and insulin (Cat#90080, Crystal Chem, Elk Grove Village, IL, USA) were determined using ELISA kits. The protocols and quantification were conducted according to the manufacturer instructions. All measurements were conducted in duplicates at the least, and the samples for which the volumes were insufficient after non-targeted metabolomic analyses were omitted from further analysis. The quantified results were analyzed by one-way ANOVA, followed by Fisher’s LSD post hoc test.

## Figures and Tables

**Table 1:** Summary of the differential model quality and marker metabolites obtained from the discovery set.

Sample Type		Plasma					Whole Brain	
Analytical Platform		GC-MS					GC-MS	LC-MS (−Mode)
Differential Model		FC vs. MC	FS vs. FC	FS vs. MS	FS vs. Others	FS vs. FC vs. MC + MS	FS vs. FC	FS vs. FC
R^2^X		0.912	0.915	0.921	0.887	0.921	0.828	0.944
R^2^Y		0.996	0.998	0.999	0.973	0.987	0.684	0.995
Q^2^		0.398	0.643	0.572	0.596	0.534	0.462	0.315
No. of features	VIP > 1.0	1643	1723	1690	1873		1009	171
	*p* < 0.05	298	490	532	610		187	35
No. of metabolites (AUC ≥ 0.7)	Total	25	27	45	35		17	6
	Identified	22	21	39	29		13	4
	Unknown	3	6	6	6		4	2

**Table 2:** Stressed female-specific plasma metabolites identified from the GC-MS analysis of discovery set.

No.	Metabolite	FS vs. FC					FS vs. MS				
		VIP	*p*	FDR	FS/FC ^d^	AUC	VIP	*p*	FDR	FS/MS ^d^	AUC
1	Pyruvic acid ^a^	- ^c^	-	-	-	-	2.74	0.00	0.04	0.65	0.89
2	L-Lactic acid ^a^	3.37	0.00	0.00	0.24	1.00	7.34	0.00	0.01	0.22	0.97
3	Glycolic acid ^a^	-	-	-	-	-	1.52	0.00	0.01	7.20	0.92
4	L-Valine ^a^	1.78	0.00	0.04	2.88	0.91	4.95	0.04	0.14	2.38	0.76
5	L-Alanine	1.22	0.03	0.14	2.01	0.81	2.11	0.03	0.11	2.02	0.70
6	3-Hydroxybutyric acid (BHB) ^a^	1.06	0.03	0.13	13.42	0.91	2.88	0.04	0.14	7.70	0.87
7	2-Hydroxy-3-methylbutyric acid ^a^	-	-	-	-	-	1.95	0.00	0.00	0.32	1.00
8	L-Isoleucine ^a^	1.70	0.03	0.12	1.41	0.78	1.10	0.02	0.11	2.90	0.79
9	Urea ^a^	3.17	0.02	0.11	0.21	0.83	3.17	0.00	0.01	0.21	0.95
10	L-Serine ^a^	3.28	0.04	0.15	2.33	0.78	1.11	0.01	0.07	3.62	0.83
11	L-Leucine ^a^	2.05	0.03	0.12	4.11	0.78	-	-	-	-	-
		-	-	-	-	-	3.24	0.04	0.15	1.19	0.78
12	Glycerol ^a^	0.01	0.03	0.12	4.11	0.78	1.21	0.00	0.01	53.08	0.86
13	L-Threonine ^a^	-	-	-	-	-	1.80	0.04	0.14	1.73	0.81
14	Glycine ^a^	-	-	-	-	-	1.23	0.03	0.13	1.96	0.73
15	Succinic acid ^a^	1.82	0.02	0.11	0.42	0.78	-	-	-	-	-
16	Fumaric acid ^a^	-	-	-	-	-	1.05	0.04	0.14	10.32	0.72
17	Aminomalonic acid ^b^	-	-	-	-	-	1.13	0.04	0.14	0.73	0.81
18	Pyroglutamic acid ^a^	-	-	-	-	-	3.11	0.04	0.13	0.71	0.78
19	L-Cysteine ^a^	1.33	0.01	0.05	1.66	0.88	1.88	0.00	0.01	2.35	1.00
20	L-Threonic acid ^b^	-	-	-	-	-	1.54	0.00	0.00	1.60	0.98
21	Oxoglutaric acid ^a^	-	-	-	-	-	2.29	0.00	0.01	10.76	0.90
22	Phenylalanine ^a^	1.27	0.01	0.08	6.11	0.81	2.70	0.00	0.02	1.33	0.89
23	L-Asparagine ^a^	-	-	-	-	-	3.99	0.01	0.05	2.61	0.86
24	L-Glutamine ^a^	-	-	-	-	-	5.82	0.00	0.01	1.35	0.91
25	Citric acid ^a^	-	-	-	-	-	4.35	0.01	0.07	3.07	0.86
26	Myristic acid ^a^	-	-	-	-	-	1.43	0.01	0.05	91.83	0.79
27	D-Pinitol ^a^	-	-	-	-	-	1.63	0.00	0.01	5.08	0.91
28	1,5-Anhydrosorbitol ^a^	1.09	0.04	0.15	1.67	0.75	1.45	0.04	0.15	1.63	0.72
29	Arabinose ^a^	-	-	-	-	-	1.28	0.02	0.09	26.91	0.79
30	Mannose ^a^	-	-	-	-	-	1.57	0.02	0.11	0.41	0.83
31	L-Tyrosine ^a^	-	-	-	-	-	1.51	0.04	0.14	1.34	0.78
32	D-Glucose ^a^	1.56	0.00	0.00	3.19	0.97	1.22	0.00	0.01	2.59	0.94
33	Palmitic acid ^a^	1.19	0.04	0.14	1.29	0.81	1.62	0.04	0.13	1.92	0.70
34	Myo-inositol ^a^	1.17	0.01	0.08	2.79	0.83	2.11	0.03	0.12	7.32	0.77
35	Linoleic acid ^a^	5.24	0.03	0.13	3.91	0.91	2.48	0.02	0.09	2.75	0.85
36	Oleic acid ^a^	-	-	-	-	-	2.13	0.02	0.11	2.76	0.82
37	Stearic acid ^a^	1.56	0.00	0.02	1.22	0.88	14.00	0.00	0.00	1.74	1.00
38	L-Cystine ^a^	2.62	0.03	0.13	1.86	0.75	-	-	-	-	-
39	Arachidonic acid ^a^	1.72	0.02	0.11	↑ ^e^	0.75	5.40	0.01	0.05	4.52	0.88
40	Oleamide ^a^	3.20	0.02	0.11	0.41	0.82	1.55	0.03	0.13	0.46	0.84
41	Cholesterol ^a^	8.23	0.00	0.00	1.21	0.95	7.00	0.00	0.04	0.86	0.92

^a^ Identified using a commercially available standard, ^b^ Identified based on literature and/or mass spectra library, ^c^ Not applicable, ^d^ Fold change, ^e^ Upregulated in comparison to zero intensity in FC.

## Data Availability

The data presented in this study are available on request from the corresponding author.

## Supplementary Materials

The supporting information can be downloaded at: https://www.mdpi.com/article/10.3390/ijms25042443/s1.
