# Supplementary material for: Non-Targeted Metabolomics Investigation of a Sub-Chronic Variable Stress Model Unveils Sex-Dependent Metabolic Differences Induced by Stress
Source: Int J Mol Sci. 2024 Feb 19;25(4):2443. doi: 10.3390/ijms25042443 (PMC10889542; doi:10.3390/ijms25042443)
Supplement: Supplementary file 1 [file ijms-25-02443-s001.zip › ijms-2871026-supplementary.pdf]

Electronic Supporting Information

Non-targeted metabolomics investigation of a sub-chronic variable stress model unveils sex-dependent metabolic differences induced by stress

Seulgi Kang [1], Woonhee Kim [2], Jimin Nam [2], Ke Li [1], Yua Kang [1], Boyeon Bae [1], Kwang-Hoon Chun [3], Chi Hye Chung [2], Jeongmi Lee [1,*]

[1] School of Pharmacy, Sungkyunkwan University, Suwon, Gyeonggi 16419, Republic of Korea

[2] Department of Biological Sciences, Konkuk University, Seoul, 05029, Republic of Korea

[3] Gachon Institute of Pharmaceutical Sciences, College of Pharmacy, Gachon University, 21936, Incheon, Republic of Korea

[*]Corresponding author: jlee0610@skku.edu. Tel.: +82-31-290-7784. Fax: +82-31-292-8800.

Experimental S1. Procedures to establish the SCVS and aLH models

For the SCVS model, both male and female were treated with three variable stressors for three days in the following order: 80 random mild foot shocks at 0.45 mA for randomized time interval (22 sec-68 sec), a tail suspension stress for 1 hour, and restraint stress for 1 hour placed inside a 50 mL conical tube within house cage. After the first cycle, the same stress sequence was repeated for the next three days. The experimental scheme is shown in Figure 1A

Experimental S2. Derivatization conditions and the order of the sample preparation and GC-MS analysis

For plasma, the samples were suspended in 150 μL of methoxyamine hydrochloride in pyridine for 90 min at room temperature and reacted with 150 μL of BSTFA for 90 min at 70 ℃. For whole brain, the samples were mixed in 150 μL of methoxyamine hydrochloride in pyridine for 90 min at 37 ℃ and reacted with 150 μL of BSTFA for 120 min at 70 ℃.

TMS derivatives of amino acids and non-amino organic may incur poor reproducibility and instability problems in GC-MS-based metabolomics analysis [17]. Indeed, stability of the derivatives at room temperature was found to vary when they were examined as a function of idling time prior to injection. Specifically, derivatives of several metabolites (e.g., glycine with two TMS groups and γ-aminobutyrate with three TMS groups) became degraded, while some (e.g., inosine with four TMS groups) underwent further derivatization over time (Figure S4). Therefore, the post-derivatization mixtures were maintained at 4 °C for no less than 2 h to prevent further reaction for sure, while the number of samples within a batch was kept as low as five to minimize the sample-to-sample variations. The optimized order of sample preparation and analysis within batch and among batches is displayed in Figure S5. In this way, most metabolite peaks showed relatively acceptable reproducibility (≤27% RSD).

Experimental S3. Analytical instruments and operating conditions for the metabolic profiling

GC-MS analysis was carried out using an 5977A coupled to 7890B mass spectrometer (full scan mode, m/z 50-800) from Agilent (Santa Clara, CA, USA). The temperatures of inlet, ion source, MS quadrupole, and MS transfer line were set at 260°C, 230°C, 150°C, and 280°C, respectively. Sample (1 μL) was injected onto an HP-5MS UI coated 5% diphenyl 95% dimethylpolysiloxane (30 m x 0.25 mm i.d.) supplied by J & W Scientific (Folsom, CA, USA) using split mode (split ratio to 10:1). For plasma analysis, the oven temperature gradient was programmed as follows: initially maintained at 80 °C for 3 min, increased to 80-250 °C at 10 °C min^{-1}, 250-300 °C at 5 °C min^{-1}, and held at 300 °C for 2 min. For brain analysis, it was programmed as follows: initially kept at 80 °C for 3 min, increased to 80-240 °C at 8 °C min^{-1}, 240-300 °C at 20 °C min^{-1}, and held at 300°C for 3 min. Post run time was set at 300°C for 5 min.

Whole brain analyses were also conducted using a Waters Xevo-G2-Q-TOF (Milford, MA, USA) operated in both negative (-) and positive (+) electrospray ionization mode in the mass range of 100-1500 m/z in continuum mode using a low collision energy and high collision energy (20 - 45 V). Five μL of sample was injected and chromatographed on an Agilent ZORBAX Eclipse Plus C18 column (100 mm × 2.1 mm, 1.8 μm) set at 40°C. The gradient elution was performed using water with 0.1% formic acid (A) and acetonitrile with 0.1% formic acid (B). The gradient condition was as follows: 0-2 min, 1% B; 2-4 min, 1-80% B; 4-8 min, 80-100% B; 8-13 min, 100% B; 13-15 min, 100-1% B; 15-17.5 min, 1% B.

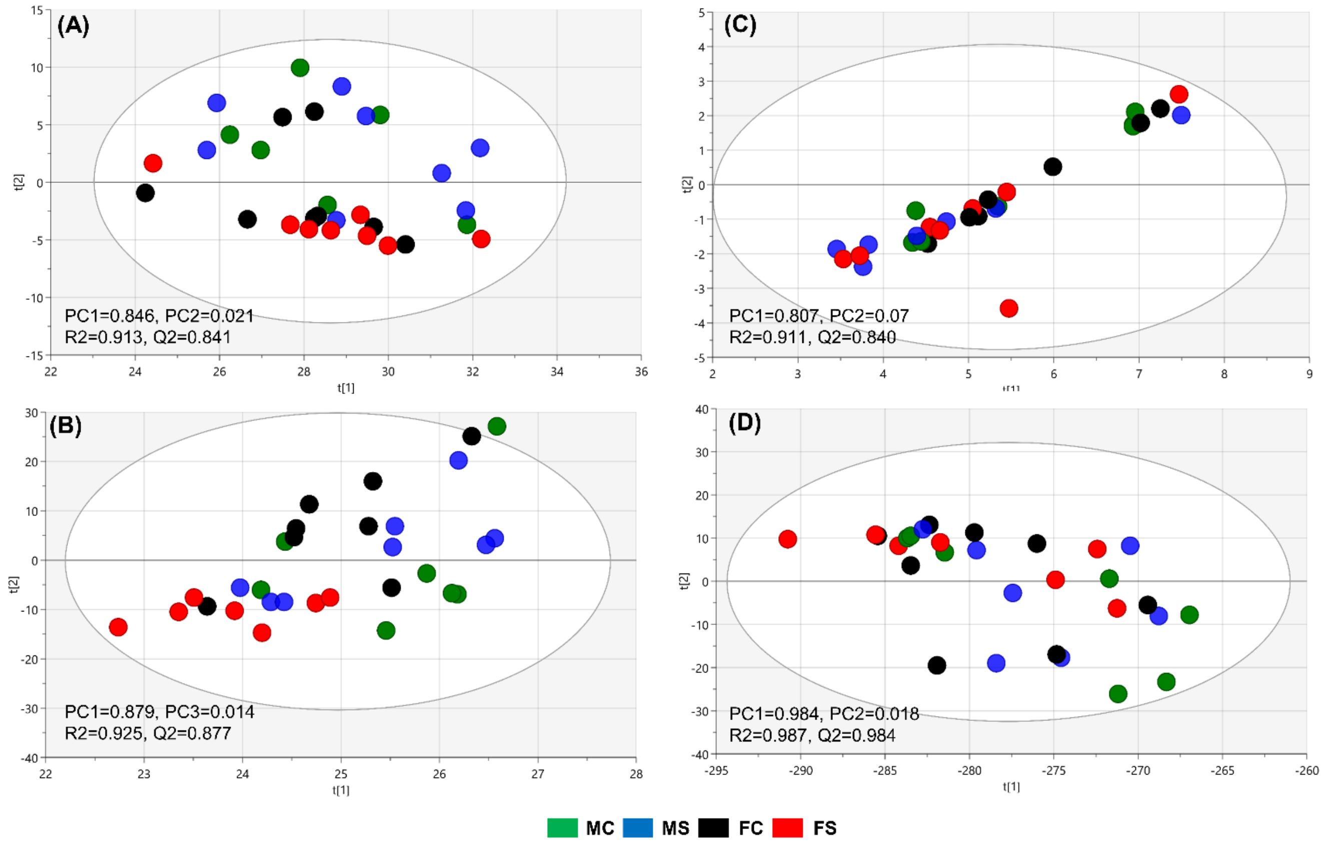

Figure S1. PCA score plots of the GC-MS-based datasets (A and B) and the LC-MS-based datasets (C and D). (A) Plasma samples, (B) brain samples, (C) brain samples obtained from the ESI- mode, and (D) brain samples from the ESI+ mode.

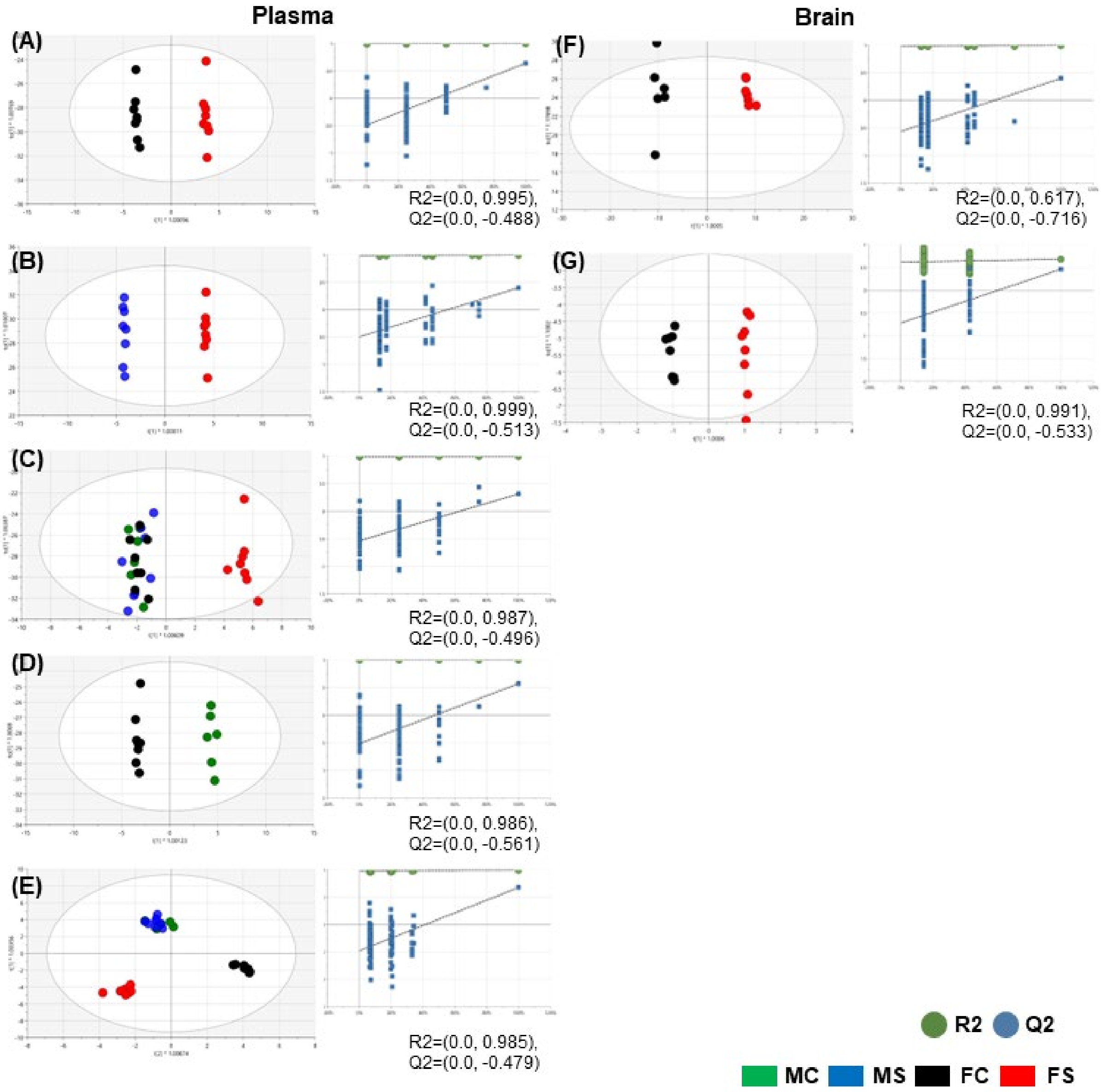

Figure S2. OPLS-DA score plot and random permutation tests with 100 iterations from the GC-MS-based metabolomes (A-F) and LC-MS (ESI-)-based metabolomes (G). (A) FS *vs*. FC, (B) FS *vs*. MS, (C) FS *vs*. Others, (D) FC *vs*. MC, (E) FS *vs*. FC *vs*. MC + MS, (F) FS *vs*. FC, (G) FS *vs*. FC.

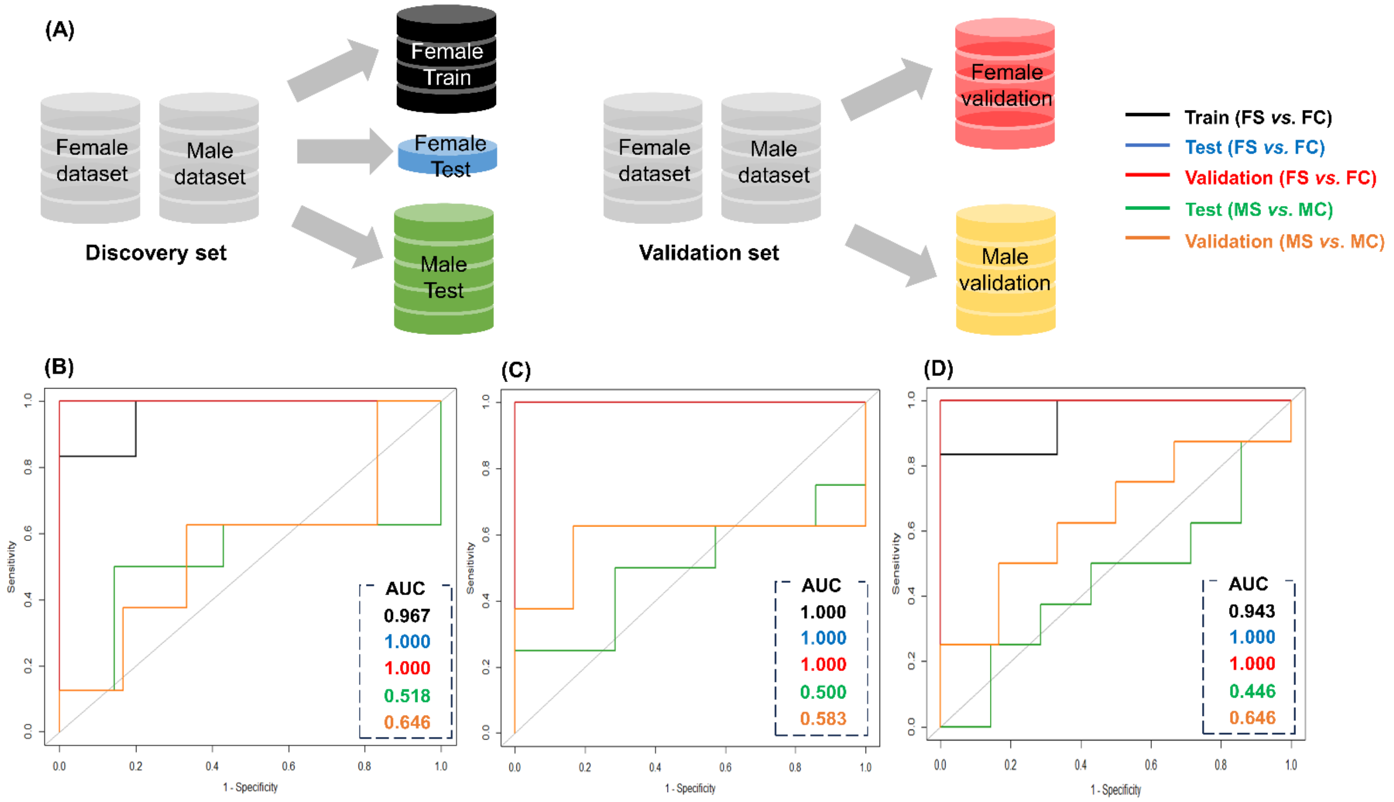

Figure S3. Data partition for the establishment and validation of biomarker panels (A) and receiver operating characteristics (ROC) curves and area under the curve (AUC) values of the biomarker panels (B-D). The used classification algorithms are support vector machine (B), random forest (C), and logistic regression (D).

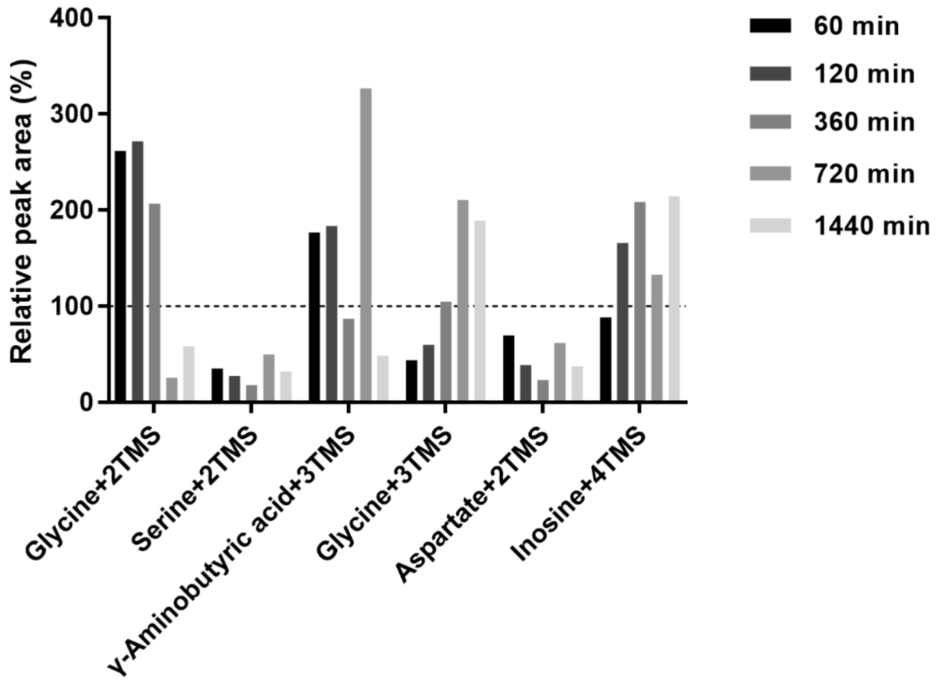

Figure S4. Room temperature stability of the derivatized metabolites from brain from 60 min to 1440 min. The peak areas of derivatized metabolites were normalized against the peak area of ribitol (internal standard) of which peak area did not change significantly (≤ 4.9 % RSD).

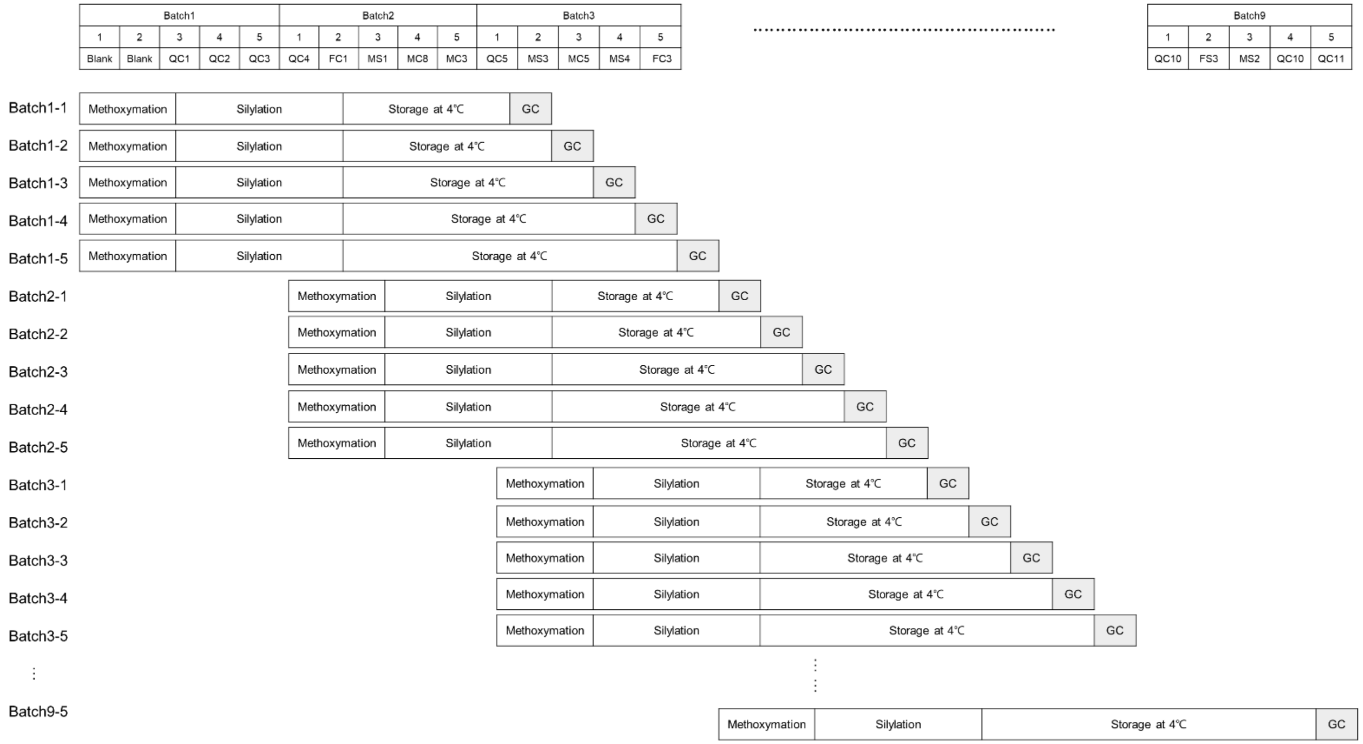

Figure S5. Order of the sample preparation and analysis by GC-MS adopted in the present study.

Table S1. Differential plasma metabolites between female (FC) and male (MC) control mice based on the GC-MS analysis of discovery set.

No.	Metabolite	TMS/MeOX	m/z	FC *vs*. MC				
				VIP	*p*	FDR	FC/MC [c)]	AUC
1	2-Linoleoylglycerol [b)]	2/0	74.1	2.55	0.00	0.00	0.34	1.00
2	L-Lactic acid [a)]	2/0	219.1	4.64	0.01	0.16	0.81	0.86
3	Glycolic acid [a)]	2/0	148.1	1.17	0.02	0.19	6.46	0.84
4	L-Alanine [a)]	2/0	100.1	1.39	0.01	0.15	1.78	0.84
5	2-Hydroxy-3-methylbutyric acid [a)]	2/0	145.1	2.43	0.00	0.00	0.25	1.00
6	Urea [a)]	2/0	59.0	2.07	0.01	0.14	0.19	0.88
7	L-Serine [a)]	2/0	73.1	3.18	0.01	0.12	3.97	0.86
8	Hydroxyproline [a)]	3/0	140.1	1.23	0.00	0.12	0.38	0.91
9	L-Cysteine [a)]	3/0	218.1	1.36	0.00	0.12	1.57	0.98
10	L-Threonic acid [b)]	4/0	292.1	1.01	0.01	0.14	1.36	0.89
11	L-Lysine [b)]	3/0	84.1	1.80	0.01	0.12	2.48	0.98
12	L-Glutamine [a)]	3/0	156.1	5.14	0.01	0.14	1.40	1.00
13	DL-Ornithine [b)]	4/0	109.1	1.16	0.00	0.11	↓ [d)]	0.93
14	Citric acid [a)]	4/0	231.1	1.05	0.01	0.12	9.65	0.86
15	D-Pinitol [a)]	5/0	174.1	1.51	0.00	0.11	↑ [d)]	0.88
16	1,5-Anhydrosorbitol [a)]	4/0	259.1	1.18	0.01	0.14	1.35	0.89
17	Mannose [a)]	5/0	75.0	2.80	0.01	0.16	7.41	0.84
18	Palmitic acid [a)]	1/0	172.0	1.07	0.01	0.16	↑	0.81
19	Linoleic acid [a)]	1/0	132.0	7.72	0.01	0.13	3.6	0.87
20	Stearic acid [a)]	1/0	202.1	10.67	0.00	0.07	1.66	1.00
21	Arachidonic acid [a)]	1/0	73.1	5.62	0.01	0.15	↑	0.81

22	Docosahexaenoic acid [b)]	1/0	73.1	4.85	0.01	0.15	6.51	0.84
23	Unknown3		83.1	1.07	0.01	0.14	↑	0.81
24	Unknown10		258.1	3.59	0.01	0.12	0.76	0.91
25	Unknown11		204.1	1.17	0.01	0.15	↑	0.81

a) Identified using a commercially available standard.

b) Identified based on literature and/or mass spectra library.

c) Fold change.

d) Up-regulated in FC compared to zero intensity in MC (↑) or zero intensity in FC compared to positive intensity in MC (↓).

Table S2. Differential brain metabolites between female stressed (FS) and control (FC) mice in the discovery set.

No.	Analytical platform	Metabolite	TMS/MeOX	m/z	FS *vs.* FC				
					VIP	*p*	FDR	FS/FC [d)]	AUC
1	GC-MS	5-Oxoproline [b)]	1/0	83.1	1.06	0.02	0.10	2.33	0.81
2		L-Aspartate [a)]	3/0	232.1	2.71	0.02	0.10	0.94	0.84
3		4-Aminobutanoate (GABA) [a)]	3/0	175.1	1.33	0.03	0.14	0.87	0.89
4		L-Glutamate [a)]	3/0	129.1	1.30	0.02	0.10	0.95	0.80
5		Glycerol-3-phosphate [b)]	4/0	299.1	2.42	0.00	0.01	0.82	0.91
6		2-Oxoglutaric acid [b)]	2/0	55.0	2.64	0.01	0.13	0.88	0.84
7		1,5-Anhydrosorbitol [a)]	4/0	217.1	1.28	0.04	0.10	1.20	0.84
8		Palmitic acid [a)]	1/0	313.2	4.03	0.03	0.14	2.92	0.80
9		Myo-Inositol [a)]	6/0	304.2	1.86	0.02	0.11	0.82	0.88
10		Oleic acid [a)]	1/0	82.1	1.42	0.02	0.09	1.18	0.83
11		Stearic acid [a)]	1/0	130.0	1.21	0.01	0.04	0.86	0.92
12		Inosine [a)]	4/0	230.1	2.40	0.03	0.14	1.21	0.81
13		Cholesterol [a)]	1/0	133.1	7.84	0.00	0.01	0.84	0.94
14		UnknownA		72.1	2.14	0.01	0.08	0.87	0.89
15		UnknownB		75.1	2.06	0.01	0.07	0.85	0.88
16		UnknownC		316.1	1.06	0.02	0.11	0.88	0.88
17		UnknownD		58.1	2.06	0.03	0.14	0.79	0.84
18	LC-MS	Adenosine monophosphate (5'-AMP) [a)]	N/A [c)]	174.0403	5.70	0.01	0.17	0.75	0.91
19		N-Acetylaspartic acid [a)]	N/A	201.0372	1.39	0.03	1.54	0.23	0.81
20		Taurine [a)]	N/A	124.0063	3.12	0.02	0.80	0.22	0.88
21		Inosine [a)]	N/A	135.0296	1.18	0.03	1.52	0.23	0.84

22	UnknownF	255.0800	1.44	0.01	0.17	1.83	0.84
23	UnknownG	236.1029	3.20	0.00	0.00	3.97	0.91

[a] Identified using a commercially available standard.

[b] Identified based on literature and/or mass spectra library.

[c] Not applicable.

[d] Fold change.

Table S3. Stressed female-specific brain metabolites from the GC-MS analysis of validation set.

No.	Metabolite	TMS/MeOX	m/z	FS *vs.* FC					FS *vs.* MS				
				VIP	*p*	FDR	FS/FC [c)]	AUC	VIP	*p*	FDR	FS/MS [c)]	AUC
1	L-Lactic acid [a)]	2/0	85.1	1.11	0.01	0.39	2.26	0.75	-	-	-	-	-
			132.0	-	-	-	-	-	5.12	0.02	0.16	0.52	0.85
2	L-Alanine [a)]	2/0	116.1	1.81	0.02	0.39	0.82	0.76	-	-	-	-	-
3	Cystathionin [b)]	2/0	73.1	1.72	0.01	0.39	4.20	0.76	1.96	0.04	0.23	2.78	0.72
4	L-Valine [a)]	2/0	73.0	-	-	-	-	-	1.17	0.02	0.16	0.82	0.79
5	Urea [a)]	2/0	173.1	1.48	0.03	0.39	1.45	0.73	2.09	0.01	0.12	1.49	0.79
6	Phosphoric acid [a)]	3/0	269.1	-	-	-	-	-	7.75	0.03	0.22	0.16	0.71
7	Glycine [a)]	3/0	248.2	2.91	0.01	0.39	↑ [d)]	0.71	-	-	-	-	-
8	L-Serine [a)]	3/0	75.1	-	-	-	-	-	2.05	0.05	0.24	3.27	0.70
9	Pyroglutamic acid [b)]	1/0	61.0	-	-	-	-	-	1.36	0.00	0.03	2.77	0.79
10	L-Aspartic acid [a)]	3/0	232.1	3.50	0.02	0.39	0.92	0.76	-	-	-	-	-
			218.1	-	-	-	-	-	1.58	0.04	0.23	0.92	0.75
11	4-Aminobutanoic acid [a)]	3/0	101.0	1.45	0.00	0.39	0.35	0.84	-	-	-	-	-
12	L-Theronic acid [b)]	3/0	205.1	6.73	0.03	0.39	2.89	0.71	-	-	-	-	-
13	L-Glutamic acid [a)]	3/0	145.0	1.22	0.02	0.39	0.30	0.76	-	-	-	-	-
			348.2	-	-	-	-	-	2.32	0.00	0.02	0.87	0.88
14	N-Acetylaspartic acid [b)]	3/0	275.1	1.41	0.05	0.41	0.59	0.74	7.41	0.00	0.00	0.56	0.90
15	Glycerol-3-phosphate [b)]	4/0	101.1	2.14	0.04	0.40	2.64	0.72	-	-	-	-	-
			103.1	-	-	-	-	-	1.53	0.04	0.23	0.73	0.72
16	Phosphoethanolamine [b)]	4/0	133.0	-	-	-	-	-	1.54	0.02	0.16	0.30	0.73
17	2-Oxoglutaric acid [b)]	2/0	55.0	3.52	0.02	0.39	0.66	0.76	2.91	0.02	0.17	0.66	0.76

18	Palmitic acid [a]	1/0	131.0	1.01	0.05	0.41	1.27	0.74	-	-	-	-	-
19	Myo-Inositol [a]	6/0	55.0	1.11	0.02	0.39	1.49	0.77	-	-	-	-	-
			113.0	-	-	-	-	-	1.08	0.01	0.11	0.73	0.81
20	Stearic acid [a]	1/0	67.0	1.03	0.03	0.39	↓ [d]		-	-	-	-	-
			59.0	-	-	-	-	-	1.13	0.02	0.16	0.43	0.77
21	2-Octadecanoylglycerol [b]	2/0	331.1	4.03	0.01	0.39	12.93	0.72	-	-	-	-	-
22	Cholesterol [a]	1/0	147.0	1.05	0.01	0.39	0.37	0.80	-	-	-	-	-
			133.0	-	-	-	-	-	1.04	0.04	0.23	0.30	0.70
23	UnknownC		132.1	-	-	-	-	-	1.90	0.04	0.23	1.99	0.70
24	UnknownD		316.1	4.32	0.05	0.41	0.68	0.73	3.57	0.04	0.23	0.69	0.72
25	UnknownE		224.1	3.29	0.02	0.39	0.54	0.78	-	-	-	-	-
			58.1	-	-	-	-	-	1.26	0.01	0.10	0.69	0.78
26	UnknownI		227.0	-	-	-	-	-	1.38	0.03	0.20	0.46	0.76
27	UnknownJ		104.1	-	-	-	-	-	1.02	0.00	0.00	8.00	0.86
28	UnknownL		147.1	1.44	0.00	0.39	0.06	0.81	2.74	0.00	0.00	0.05	0.78
29	UnknownM		70.1	1.09	0.03	0.39	2.38	0.74	1.51	0.05	0.25	2.64	0.71

[a] Identified using a commercially available standard.

[b] Identified based on literature and/or mass spectra library.

[c] Fold change.

[d] Up-regulated in FS compared to zero intensity in FC (↑) or zero intensity in FS compared to positive intensity in FC (↓).

Table S4. Stressed female-specific plasma metabolites from the GC-MS analysis of validation set.

No.	Metabolite	TMS/MeOX	m/z	FS *vs*. FC					FS *vs*. MS				
				VIP	*p*	FDR	FS/FC [c)]	AUC	VIP	*p*	FDR	FS/MS [c)]	AUC
1	Pyruvic acid [a)]	1/1	115.0	-	-	-	-	-	1.76	0.02	0.24	↑	0.75
2	L-Lactic acid [a)]	2/0	131.0	-	-	-	-	-	3.16	0.02	0.24	1.18	0.85
3	Glycolic acid [a)]	2/0	66.0	-	-	-	-	-	1.11	0.04	0.25	2.64	0.89
4	L-Valine [a)]	2/0	73.1	-	-	-	-	-	3.43	0.04	0.25	2.56	0.79
5	2-Hydroxybutyric acid [b)]	2/0	131.1	1.39	0.04	0.29	1.98	0.83	-	-	-	-	-
6	3-Hydroxybutyric acid (BHB) [a)]	2/0	76.0	-	-	-	-	-	1.63	0.01	0.21	9.15	0.81
			99.0	1.46	0.03	0.25	11.58	0.87	-	-	-	-	-
7	2-Hydroxy-3-methylbutyric acid [a)]	2/0	145.1	-	-	-	-	-	2.17	0.00	0.00	0.34	1.00
8	Urea [a)]	2/0	79.1	7.32	0.02	0.21	↑ [d)]	0.79	-	-	-	-	-
			134.0	-	-	-	-	-	1.06	0.02	0.24	1.21	0.79
9	L-Serine [a)]	2/0	74.0	1.97	0.02	0.21	2.62	0.83	-	-	-	-	-
		3/0	224.0	-	-	-	-	-	3.13	0.03	0.24	9.16	0.90
10	Glycerol [a)]	3/0	74.1	1.83	0.00	0.05	1.51	0.97	-	-	-	-	-
11	Phosphoric acid [a)]	3/0	301.0	1.21	0.03	0.22	0.36	0.87	1.12	0.00	0.08	0.36	0.94
12	L-Threonine [a)]	2/0	75.0	-	-	-	-	-	1.64	0.03	0.24	2.57	0.89
		3/0	128.1	-	-	-	-	-	1.08	0.03	0.24	8.49	0.79
13	Glyceric acid [b)]	3/0	189.0	-	-	-	-	-	1.03	0.00	0.11	2.95	0.96
14	Malic acid [a)]	3/0	55.0	1.25	0.03	0.21	0.46	0.90	-	-	-	-	-
15	L-Methionine [b)]	2/0	176.0	3.35	0.02	0.21	0.00	0.90	-	-	-	-	-
16	Pyroglutamic acid [a)]	2/0	73.0	-	-	-	-	-	2.38	0.01	0.20	1.60	1.00
17	L-Cysteine [a)]	3/0	218.1	1.03	0.02	0.21	0.63	0.93					

18	L-Threonic acid [b)]	4/0	292.1	-	-	-	-	-	1.95	0.01	0.21	1.47	0.88
19	L-Glutamate [a)]	3/0	303.1	3.29	0.02	0.21	0.09	0.85	-	-	-	-	-
20	Phenylalanine [a)]	2/0	74.0	1.13	0.02	0.21	0.15	0.88	-	-	-	-	-
21	L-Glutamine [a)]	3/0	129.1	-	-	-	-	-	1.99	0.01	0.20	4.42	0.84
22	Citric acid [a)]	4/0	465.2	-	-	-	-	-	1.06	0.01	0.19	0.53	0.88
23	Myristic acid [a)]	1/0	117.0	1.91	0.01	0.21	8.77	0.87	-	-	-	-	-
24	1,5-Anhydrosorbitol [a)]	4/0	116.1	1.18	0.00	0.08	1.29	0.97	1.16	0.00	0.09	1.21	0.92
25	D-Glucose [a)]	5/0	362.2	2.14	0.02	0.21	4.49	0.92	-	-	-	-	-
		5/0	76.1	-	-	-	-	-	1.50	0.04	0.25	2.61	0.79
26	Palmitoleic acid [a)]	1/0	311.2	3.11	0.00	0.08	3.24	0.97	-	-	-	-	-
27	Palmitic acid [a)]	1/0	53.0	-	-	-	-	-	1.06	0.00	0.07	5.66	0.88
		1/0	313.2	4.39	0.02	0.21	1.36	0.80	-	-	-	-	-
28	Myo-inositol [a)]	6/0	443.2	6.25	0.02	0.21	14.24	0.90	-	-	-	-	-
29	Oleic acid [a)]	1/0	74.0	1.43	0.01	0.11	1.89	1.00	1.41	0.01	0.23	2.28	0.94
30	Stearic acid [a)]	1/0	129.0	3.78	0.02	0.21	2.64	0.93	2.88	0.02	0.24	1.44	0.90
31	2-Octadecanoylglycerol [b)]	2/0	399.3	1.11	0.02	0.21	0.78	0.93	-	-	-	-	-
32	Unknown3		75.0	-	-	-	-	-	1.42	0.01	0.19	2.79	0.85
33	Unknown4		226.0	-	-	-	-	-	4.24	0.02	0.24	↑	0.83
			59.0	1.60	0.07	0.00	↑	0.83	-	-	-	-	-
34	Unknown6		362.1	2.14	0.02	0.21	4.49	0.87	-	-	-	-	-
			332.1	-	-	-	-	-	2.44	0.01	0.19	↑	0.83
35	Unknown12		227.1	-	-	-	-	-	4.04	0.00	0.00	8.40	1.00
36	Unknown13		117.0	1.54	0.02	0.21	0.00	0.80	-	-	-	-	-

[a)] Identified using a commercially available standard. [b)] Identified based on literature and/or mass spectra library. [c)] Fold change. [d)] Up-regulated in FS compared to zero intensity in FC (↑).

Table S5. Diagnostic performance of the biomarker panels consisting of BHB, myo-inositol, L-serine, and glycerol.

Algorithm	Biological data	Dataset	Sex	Accuracy	Precision	Sensitivity	Specificity	(95% CI)	TP	FP	FN	TN
Support vector machine	Discovery set	Training	F	0.818	1.000	0.600	1.000	(0.4822, 0.9772)	3	0	2	6
		Test	F	1.000	1.000	1.000	1.000	(0.4782, 1)	3	0	0	2
			M	0.667	0.600	0.857	0.500	(0.3838, 0.8818)	6	4	1	4
	Validation set	Validation	F	1.000	1.000	1.000	1.000	(0.7151, 1)	5	0	0	6
			M	0.500	0.429	0.500	0.500	(0.1766, 0.7114)	3	4	3	4
Random forest	Discovery set	Training	F	1.000	1.000	1.000	1.000	(0.7151,1)	6	0	0	6
		Test	F	0.750	0.667	1.000	0.500	(0.1941,0.9937)	2	1	0	1
			M	0.533	0.500	1.000	0.125	(0.2659,0.7873)	7	7	0	1
	Validation set	Validation	F	1.000	1.000	1.000	1.000	(0.7151,1)	5	0	0	6
			M	0.500	0.444	0.667	0.375	(0.2304,0.7696)	4	5	2	3
Logistic regression	Discovery set	Training	F	1.000	1.000	1.000	1.000	(0.7354, 1)	6	0	0	6
		Test	F	0.750	0.500	1.000	0.667	(0.1941, 0.9937)	1	1	0	2
			M	0.600	0.875	0.546	0.750	0.3229, 0.8366)	6	1	5	3
	Validation set	Validation	F	0.909	1.000	0.833	1.000	(0.5872, 0.9977)	5	0	1	5
			M	0.429	0.500	0.375	0.500	(0.1766, 0.7114)	3	3	5	3
